# Long Non-Coding RNAs in Metabolic Organs and Energy Homeostasis

**DOI:** 10.3390/ijms18122578

**Published:** 2017-11-30

**Authors:** Maude Giroud, Marcel Scheideler

**Affiliations:** 1 Institute for Diabetes and Cancer (IDC), Helmholtz Zentrum München, German Research Center for Environmental Health, 85764 Neuherberg, Germany; maude.giroud@helmholtz-muenchen.de; 2 Joint Heidelberg-IDC Translational Diabetes Program, Heidelberg University Hospital, 69120 Heidelberg, Germany; 3 Germany Center for Diabetes Research (DZD), 85764 Neuherberg, Germany

**Keywords:** non-coding RNA, lncRNA, metabolic organs, liver, pancreas, skeletal muscle, cardiac muscle, adipose tissue, metabolism, energy homeostasis

## Abstract

Single cell organisms can surprisingly exceed the number of human protein-coding genes, which are thus not at the origin of the complexity of an organism. In contrast, the relative amount of non-protein-coding sequences increases consistently with organismal complexity. Moreover, the mammalian transcriptome predominantly comprises non-(protein)-coding RNAs (ncRNA), of which the long ncRNAs (lncRNAs) constitute the most abundant part. lncRNAs are highly species- and tissue-specific with very versatile modes of action in accordance with their binding to a large spectrum of molecules and their diverse localization. lncRNAs are transcriptional regulators adding an additional regulatory layer in biological processes and pathophysiological conditions. Here, we review lncRNAs affecting metabolic organs with a focus on the liver, pancreas, skeletal muscle, cardiac muscle, brain, and adipose organ. In addition, we will discuss the impact of lncRNAs on metabolic diseases such as obesity and diabetes. In contrast to the substantial number of lncRNA loci in the human genome, the functionally characterized lncRNAs are just the tip of the iceberg. So far, our knowledge concerning lncRNAs in energy homeostasis is still in its infancy, meaning that the rest of the iceberg is a treasure chest yet to be discovered.

## 1. Introduction

### 1.1. Impact of Non-Protein Coding RNA from a Genomic Point of View

For a long time, the number of protein coding genes has been used to determine the complexity of an organism. That implies that the amount of DNA should be higher in a more complex organism. However, several discrepancies have been identified: sequencing of entire genomes surprisingly revealed (i) that cells of some salamanders may contain 40 times more DNA than those of humans [1] and (ii) that some single cell organisms such as *Tetrahymena thermophila* even exceed the number of human protein coding genes [2]. Interestingly, the analysis of sequenced genomes demonstrates that the relative amount of non-protein-coding sequences increases consistently with organismal complexity. This relationship suggests that those non-coding elements exert a function that would require transcriptional activity. This has been investigated by the ENCODE project, which elucidated that 74.7% of the human genome is indeed covered by primary transcripts that predominantly represent non(-protein)-coding RNAs (ncRNAs) [3,4].

### 1.2. Classification of ncRNAs

Researchers have arbitrarily divided ncRNAs in two groups: small (<200 nucleotides (nt)) and long (>200 nt) non-coding RNAs, which can be classified as housekeeping or regulatory transcripts, respectively. The small ncRNAs comprise transfer RNAs (tRNAs), small nucleolar RNAs (snoRNAs), and small nuclear RNAs (snRNAs) as housekeeping RNAs, while piwi protein-associated RNAs (piRNAs) and microRNAs (miRNAs) are regulatory RNAs. On the other hand, non-coding RNAs larger than 200 nt are represented by housekeeping RNAs like ribosomal RNAs (rRNAs), while long non-coding RNAs (lncRNAs) are regulatory elements, including antisense RNAs (AS-RNA) and enhancer RNAs (eRNAs) [5,6,7]. 

In order to distinguish lncRNAs from protein-coding transcripts, lncRNAs are characterized by their length, their intron/exon structure, the presence of a 3′ UTR and termination region, and their limited coding potential supported by the absence of ORFs. lncRNAs are expressed at lower levels than mRNAs, often in a species- and tissue-specific manner [8,9,10,11]. Withal, as mRNAs, lncRNAs are transcribed by the RNA polymerase (POL) II [12], spliced [13], polyadenylated [14], and capped at the 5′ end [15]. Moreover, during the last decade, the role of lncRNAs in epigenetics has been largely investigated elucidating a tremendous variety of mechanisms of action such as gene expression regulation (signal lncRNAs), histone modification (scaffold lncRNAs), recruitment of chromatin modifying enzymes (guide lncRNAs), and titration of transcriptional factors and miRNAs (decoy lncRNAs) [16]. Of note, this large panel of activities has also been linked to a great number of pathophysiological conditions, including metabolic diseases such as cardiovascular disease, diabetes, and obesity.

### 1.3. lncRNAs’ Modes of Action

lncRNAs are found everywhere in the genome: from enhancer sequences, promoter regions, 5′ UTRs, exons, introns, intragenic regions, intergenic sequences, antisense sequences, and 3′ UTRs. In the same way, as protein-coding RNA, lncRNAs may also be subjected to splicing and post-transcriptional epigenetic modifications such as histone 3 lysine 4 trimethylation (H3K4me3). lncRNAs are poorly conserved between species and are highly tissue-specific [17], which makes them very specific and tightly regulated, even though they are found at lower levels compared to mRNAs [9,10,11,12].

The regulatory role of lncRNAs depends directly on their cellular localization [18]. In the nucleus, lncRNAs can act as transcriptional activators or inhibitors in *cis* (regulating neighboring genes) or in *trans* (regulating genes from other regions or chromosomes). In the cytoplasm, lncRNAs have been shown as molecular decoys for proteins or microRNAs. lncRNAs’ mode of action is versatile due to their ability to bind a large spectrum of molecules like DNA, RNA, and protein [19]. For example, the lnc-SRA is a chromatin regulator via enhancing insulator function of CCCTC-binding factor (CTCF) [20], while HOTAIR targets the lysine specific demethylase 1A (Lsd1) complex to demethylate H3K4me2 [21]. lncRNAs can also regulate transcription factor activity. For example, growth arrest specific 5 (Gas5) regulates steroid receptor (SR) activity by titrating its own glucocorticoid receptor (GR) binding site against genomic GR binding sites [22]. Another activity of lncRNAs is the degradation of mRNA targets (lincRNA-p21 on JunB proto-oncogene (JUNB) mRNA, or 1/2-sbsRNAs on staufen 1 (STAU1)-mediated messenger RNA decay mRNA [23]). As an example of a lncRNA that binds proteins, MALAT1 modulates SR protein splicing factor phosphorylation and thus downstream target splicing [24]. Very recently, the lncRNA NRB2 has been described as directly binding AMPk and inducing its activation in the context of energy stress in cancer cells. Those studies are the first to report a direct lncRNA–kinase interaction [25,26,27]. lncRNAs are also a reservoir of small ncRNAs such as H19 or HULK, which respectively serve as a source for microRNAs miR-675 and miR-37 [28]. Moreover, they can regulate those microRNAs and others coming from distant loci by acting as a decoy, thus preventing microRNAs from binding to and blocking their target mRNAs. In rare cases, lncRNAs present putative open reading frames and actually can encode peptides as small regulatory polypeptides of amino acid response (SPAR) encoded from LINC00961 [29]. Cytoplasmic lncRNAs seem to bind to the ribosome where they are degraded [30].

### 1.4. Metabolic Flexibility and Energy Homeostasis

Metabolism is characterized by (i) the conversion of food to energy for running cellular processes; (ii) the usage of nutrients to build nucleic acids, lipids, proteins, and carbohydrates; and (iii) the elimination of nitrogenous waste. The achievement of those functions requires a constant crosstalk between organs, tightly fine-tuned and spatially regulated. Usually, the organism responds to internal or external perturbations and adapts energy production and storage. To maintain homeostasis, cells refer to numerous metabolic pathways able to transduce a signal from metabolic sensors via a large network of signaling cascades. 

The most common metabolic challenges are nutrient overload, deprivation, and maintenance of body temperature. To address these challenges, for example, the adipose organ has a tremendous plasticity by either storing energy as lipids upon excess food intake, providing energy upon starvation, or producing heat for defending body temperature upon cold exposure [31,32,33]. 

The signaling behind the energy homeostasis has been extensively investigated regarding transcription factor and/or microRNA regulation [34,35]. Despite the fact that the first lncRNAs H19 and Xist have already been discovered in 1991 [36,37], the last 10 years significantly contributed to our knowledge of lncRNAs in metabolism, supported by achievements of the ENCODE project [3,4] and functional annotation of the mammalian genome (FANTOM) [38,39]. Nevertheless, the lncRNA research field is still in its infancy, with many discoveries to be expected in the near future. 

### 1.5. Energy Homeostasis and Disease 

Metabolic diseases occur when the body’s usual metabolic processes are disturbed or disrupted. They affect metabolic organs such as the liver, adipose, muscle, pancreas, heart, and brain. The concept of metabolic flexibility is defined by the capability of an organism to adapt fuel usage to changes in availability and is thus a key determinant of functional energy homeostasis [40]. When nutrients are not limited, excess energy can be stored in the form of triglycerides mainly in the adipose organ. In times of starvation, this stored energy can be released by inducing lipolysis to make fatty acids available to meet the energy demands of peripheral tissues [41,42]. These days, nutrients are more and more available in excess, and the sedentary lifestyle becomes more and more prevalent. Both have led to an imbalance between energy uptake and expenditure. The consequence results in an alarming high prevalence of obesity and overweight worldwide, often associated to other diseases such as type 2 diabetes (T2D), low grade inflammation, cancer, and cardiovascular disease [43,44].

It is nowadays widely accepted that the obesity pandemic is caused by a combination of external (nutrition, sedentary lifestyle, etc.) and internal (genetic) components. To respond to these perturbations, cells use epigenetic regulation to modulate the expression of their genome. lncRNAs are at the center of this epigenetic regulation and add another regulatory layer in biological processes and pathophysiological conditions. In this respect, we will summarize here the role of lncRNAs in different metabolic organs in order to better understand how lncRNAs can be principal actors of metabolic flexibility.

## 2. lncRNAs in Metabolic Organs

lncRNAs have been studied in many diseases including cancer [28,45,46], cardiovascular diseases [47,48], diabetes, and obesity [49]. In this review, we will summarize lncRNAs affecting metabolic organ homeostasis with a focus on thermogenesis (see also Table 1).

### 2.1. lncRNAs in Liver

Liver is one of the key organs for energy homeostasis, centered at the metabolic crosstalk between different organs including adipose tissues and skeletal muscle. One physiological role of liver is to balance the amount of glucose that is available in circulation. To simplify, glucose enters the glycolysis pathway where it is transformed into pyruvate. The liver uses pyruvate to generate ATP through the TCA cycle and oxidative phosphorylation in the mitochondria. Its activity is controlled by several metabolic hormones including insulin. Obesity leads to an increase of the energy available in the liver which promotes insulin resistance. Dysregulation of energy metabolism in the liver promotes metabolic syndrome-associated diseases such as non-alcoholic fatty liver diseases (NAFLD), hyperglycemia, dyslipidemia, and even diabetes [50].

lncRNAs are implicated in liver metabolism and energy homeostasis. lncLSTR (liver specific triglyceride regulator) was the first liver-enriched lncRNA to be described as regulating liver glucose and lipid metabolism. The depletion of lncLSTR in mouse liver induces an increased clearance of triglycerides (TG) dependent on an upregulated expression of apolipoprotein C-II (apoC2). lncLSTR directly binds transactive response (TAR) DNA-binding protein (Tdp-43) and forms a molecular complex to regulate the expression of the hepatic cytochrome P450 (Cyp8b1) that induces ApoC2 expression through farnesoid X receptor (Fxr). In a hyperlipidemic mouse model, lncLSTR depletion can reduce blood glucose and triglyceride levels [51]. In a further study, APOA1-AS (antisense transcript of Apolipoprotein A-I (ApoA1), a major protein component of high-density lipoprotein (HDL) in plasma), was described as an inhibitor of ApoA1 expression leading to low HDL cholesterol concentration, thus increasing the susceptibility to atherosclerosis. APOA1-AS can modulate distinct histone methylation patterns that mark active and/or inactive gene expression through the recruitment of histone-modifying enzymes to the APO cluster. APOA1-AS inhibition enhances ApoA1 protein expression in vitro in hepatic cell lines and in vivo in African grey monkeys. The authors claim that the results demonstrate a therapeutic potential for non-coding transcripts [52]. The lncRNA SRA (steroid receptor RNA activator) was shown to promote hepatic steatosis via repression of adipose triglyceride lipase (Atgl) by inhibiting transcriptional activity of forkhead box protein O1 (FoxO1). The final observation was a decrease of hepatic free fatty acid β-oxidation [53]. Another lncRNA, MALAT1, has been described to promote hepatic steatosis and insulin resistance via stabilization of nuclear Srebp-1C [54]. In fasting conditions, lncLGR is increased and specifically binds to heterogeneous nuclear ribonucleoprotein L (hnRNPL), a transcriptional repressor of glucokinase (GCK). Therefore, lncLGR knockdown enhances GCK expression and glycogen storage in fasted mice [55]. Very recently, the RNA sequencing of db/db mouse liver complemented with in vitro work highlighted the role of lncRNA H19 in hepatic physiology during diabetes. Inhibition of H19 significantly increases the levels of several gluconeogenic genes in vitro, impairs insulin signaling, and increases the nuclear localization of FoxO1 [56]. 

To conclude, despite the fact that the studies about lncRNAs in liver metabolism are not older than three years, it is without any doubt that glucose and lipid metabolism is epigenetically affected by lncRNA regulation in the liver. 

### 2.2. lncRNAs in Pancreas

The pancreas is an endocrine organ in direct connection to the liver, spleen, and small intestine. For example, the pancreas secretes digestive enzymes such as pancreatic lipases, amylases, nucleases, and phospholipases by acinar cells, which represent 98% of the total pancreatic mass. The other 2% of the pancreas are islets of Langerhans, which are able to sense alterations in circulating levels of macronutrients and to secrete hormones to govern blood glucose levels in order to maintain energy homeostasis. In healthy individuals, nutrient ingestion leads to higher circulating glucose levels, which trigger the secretion of insulin by β-cells. On the contrary, fasting leads to the production and secretion of glucagon by α-cells to promote gluconeogenesis and glycogenolysis. In case of disbalanced metabolism via elevated levels of circulating nutrients, β-cell mass is extended to compensate for the elevated needs of insulin. In certain cases of obesity, due to insulin resistance, elevated production and secretion of insulin is often not sufficient to thwart glucagon secretion [57].

In 2012, a study by Móran et al. [58] demonstrated that more than 1100 lncRNAs exist in human pancreatic islets and purified β-cells. However, the functional characterization of these pancreatic lncRNAs with an impact on energy homeostasis and Langerhans islet activity has just begun [59,60]. The first regulatory pancreatic lncRNA, HI-LNC25, has an impact on Glis Family Zinc Protein 3 (Glis3), which encodes a transcription factor that has been shown to be mutated in a form of monogenic diabetes and to contain T2D risk variants [58]. Another study showed that HI-LNC25 is necessary for proper specification and function of endocrine cells through the regulation of islet-specific transcription factors located nearby the βlinc1 locus. In line with that, HI-LNC25 deletion resulted in defective islet development and disrupted glucose homeostasis [61]. The pancreatic lncRNA TUG1 (expressed in NIT-1 cells) has been shown to be dynamically regulated by glucose, and knockdown of TUG1 resulted in increased apoptosis leading to decreased islet mass and insulin secretion in β-cells both in vitro and in vivo. These data have been corroborated with immunohistochemistry analyses [62]. Moreover, the lncRNA PLUTO has been described to regulate a β-cell-specific transcription factor network by affecting the 3D chromatin structure and consequently the transcription of pancreatic and duodenal homeobox 1 (Pdx1) [63]. Finally, lncRNA MEG3 has been reported as an integrative factor of islet secretion, with decreased expression levels in islets upon type 1 (T1D; NOD female mice) and type 2 (T2D; db/db mice) diabetes. MEG3 expression is modulated by glucose, and, as MEG3 depletion promotes β-cell apoptosis, probably via Pdx1 and MafA, it impairs insulin synthesis and secretion [64].

To conclude, β-cell metabolism has largely been investigated highlighting the impact of several factors differentially regulated in β-cells upon diabetes. The past three years have yielded great success in understanding pancreatic lncRNAs related to metabolism, also in particular with impact on β-cell specific transcription factor regulation, consequently contributing to appropriate regulation of the β-cell endocrine function.

### 2.3. lncRNAs in Skeletal Muscle

Skeletal muscles are well known to function in shaping and moving the body and represent on average 40% of the whole body mass. Importantly, skeletal muscle is a metabolic organ of high metabolic activity regarding nutrient storage and supply and its ability to answer to hormonal stimuli such as insulin and catecholamine [65,66,67]. In the postprandial stage, skeletal muscle is responsible for taking up nearly 80% of glucose in response to insulin [68,69]. During powerful and rapid contraction, fast-twitch fibers are fueled by anaerobic metabolism producing energy via glycolysis. A side effect is lactate, which is released to the bloodstream, later converted to glucose in the liver by gluconeogenesis (Cori Cycle). During prolonged energy demands, slow-twitch fibers are the most requested ones which switch to aerobic metabolism mainly fueled by β-oxidation of fatty acids. In addition, skeletal muscle plays a central role in temperature homeostasis and can produce heat through shivering, non-shivering thermogenesis [70,71], and diet induced thermogenesis [71,72]. In T2D, energy homeostasis is imbalanced leading to lipid storage in adipose tissue but also ectopically in liver and muscle [73]. Upon obesity, reduced muscular oxidative capacity as well as elevated production of reactive oxygen species (ROS) in mitochondria has been correlated with the development of insulin resistance [74,75].

Numerous lncRNAs have been related to skeletal muscle development and differentiation. Among them the lncRNA MUNC, transcribed upstream of MyoD, plays an enhancer role in transcription of MyoD, while MUNC inhibition blocked myoblast differentiation [75]. A family of lncRNAs named YAMS (YY1-associated muscle lncRNAs) has also been related to the transcriptional regulation of skeletal muscle genes. YAMS can either promote myogenesis (Yam-2 and Yam-3) or impair muscle differentiation (Yam-1 and Yam-4). Interestingly, Yam-1 has been described to affect the expression of the neighboring gene coding for miR-715, which is known to target and repress Wnt7b in skeletal muscle, a positive regulator of myogenesis [76]. The lncRNA H19 is enriched in skeletal muscle and encodes two miRNAs, miR-675-3p and miR-675-5p. H19-deficient mice have an impaired muscle regeneration after injury, which can be remedied by reintroduction of both hosted miRNAs [77]. Other lncRNAs govern muscle differentiation by functioning as molecular decoys, such as lncRNA-MD1 which is involved in myocyte differentiation by titrating miR-133 and miR-135. Both miRNAs govern the expression of two essential regulators of myogenic differentiation, myocyte enhancer factor 2C (Mef2c) and mastermind-like protein 1 (Maml1) [78]. Furthermore, linc-MD1 has been described to be implicated in myogenesis in a positive feedforward loop with Hu-antigen R (HuR), which controls biogenesis of miR-133b and its host linc-MD1. In addition, this study also demonstrated the need for a timely controlled expression of linc-MD1 and HuR in order to allow correct progression of muscle differentiation, at least in vitro [79].

Numerous lncRNAs have also been associated with diseases such as muscular dystrophy. For example, linc-MD1 has been described to be greatly reduced in muscles of patients with Duchenne muscular dystrophy (DMD), while linc-MD1 overexpression can promote myogenic differentiation [78]. In facioscapulohumeral muscular dystrophy (FSHD), local chromatine decompaction allows lncRNA-DBE-T transcription and therefore the recruitment of trithorax group protein of histone methyltransferase Ash1L, leading to activation of FSHD candidate genes [80]. More recently, the lncRNA linc-RAM has been described to be upregulated during myogenesis, to directly bind the myogenic regulatory factor MyoD, and to facilitate the assembly of the MyoD/Baf60c/Brg1 complex. In vivo, knockout of linc-RAM led to disturbed muscle regeneration [81]. 

All together, these studies provide evidence that muscular lncRNAs have a regulatory impact on muscle differentiation with consequences on muscle physiology and disease. However, studies on lncRNAs that govern skeletal muscle metabolism and/or thermogenesis have been lacking. Thus more remains to be discovered for muscular lncRNAs in order to be able to draw a more comprehensive picture of the regulatory network in skeletal muscle metabolism.

### 2.4. lncRNAs in Cardiac Muscle

The cardiac muscle is permanently exposed to tissue-extrinsic (peptide, hormones, etc.) as well as tissue-intrinsic (intracellular calcium, nutrient availability, etc.) signals. For example, leptin regulates function and metabolism of the heart. In addition, imbalanced calcium homeostasis affects cardiomyocyte fibrosis/diastolic dysfunction [82]. Moreover, the cardiac muscle is also able to secrete hormones such as oxytocin and natriuretic peptide [83]. In the context of diabetes, the early stages of diabetic cardiomyopathy are characterized by diastolic dysfunction and ventricular hypertrophy. Later on, systolic dysfunction progresses to decompensated heart failure. These symptoms can develop independently of other risk factors such as coronary diseases. In diabetic patients, insulin resistance alters cardiac metabolism by enhancing PPARa activation. Cardiac muscle is a highly active metabolic organ characterized by its plasticity and its capability of remodeling upon stress. In order to prevent heart failure, it is of interest to understand how those rearrangements are governed [84]. 

An inventory of lncRNAs regulated in cardiac remodeling can be found in a review by Shen et al. (lncRNA P21, lncRNA APF, lncRNA CARL, lncRNA Chaer, lncRNA CHRF, lncRNA H19, lncRNA Mhrt, lncRNA MIAT, lncRNA NRF, and lncRNA ROR) [47]. Other lncRNAs are necessary for the appropriate development of the cardiac organ. For example, lncRNA BVHT, mainly expressed in the heart, is required for the differentiation of progenitors from the primary germ layer mesoderm into cardiomyocytes. BVHT binds polycomb repressive complex 2 (PRC2) component Suz12 to activate a cardiovascular signaling cascade via epigenetic regulation of MesP1 [85]. Very recently, the lncRNA UPH (or heart- and neural crest derivatives-expressed 2 (Hand2)-AS1 in human) has also been related to cardiac development via *cis* regulation of the downstream transcription factor Hand2. Interestingly, that lncRNA itself is not the main actor of regulation, it is indeed the transcription of lncRNA UPH, which is necessary for Hand2 expression via RNA POL II recruitment [86]. Furthermore, the lncRNAs 1/2-sbsRNA (intermolecular base-pairing between SINE-containing lncRNAs) are part of a lncRNA family necessary for myogenesis. For example, lncRNA 1/2-sbsRNA B2 has been shown to inhibit myogenesis via downregulating TNF receptor-associated factor 6 (Traf6) [23,87]. 

To conclude, lncRNAs have emerged to be implicated in various processes of myocytes ranging from development to apoptosis. Without surprise, lncRNAs are also part of the remodeling process and implicated in several precursor symptoms of heart failure such as cardiac hypertrophy, post-ischemic remodeling, and vascular remodeling. However, studies on lncRNAs that also govern cardiac muscle metabolism have been lacking and would be an interesting field of research.

### 2.5. lncRNAs in Brain

The brain is a key organ in the regulation of energy homeostasis, able to sense levels of metabolic inputs like hormones and nutrients and able to regulate food intake and energy expenditure [88]. For example, insulin and leptin receptors are highly expressed in the hypothalamus, mediating the signaling of hunger or satiety. It has been shown in rodents that lesions in the hypothalamus impact body weight, suggesting that neuronal regeneration is of major importance for energy homeostasis [89]. 

lncRNAs are implicated in this process. For example, the lncRNA MIAT affects brain development via aberrant splicing of Wnt7b [90]. Moreover, lncRNAs are also affected and involved in neurodegenerative diseases that perturb brain function and are inter alia the main result of cell degeneration. For example, MALAT1 and NEAT1 are bound by the Huntington’s disease induced genes, TDP-43 and FUS, which make them important actors in synapse formation. NEAT1 is upregulated in the brain of patients with Huntington’s disease [91,92]. lncRNA BACE1-antisense (BACE1-AS) levels were found to significantly increase in Alzheimer brains together with mRNA and protein BACE1 levels. Moreover, BACE1-AS act as a sponge on miR-545-5p and stabilizes BACE-1 mRNA levels to raise αβ peptide production [93]. A recent study compared lncRNA expression in hypothalamus of mice following either a 24 h fast or ad libitum access to food. Six hundred twenty-two lncRNAs differentially regulated have been identified traducing the high potential of lncRNAs to be regulated by the environment [94]. Nevertheless, no extensive analysis has been yet performed on those lncRNAs.

Although lncRNAs have been found to be also involved in brain development and disease, the impact of neuronal lncRNAs on metabolism is still an open field for research.

### 2.6. lncRNAs in the Adipose Organ

The adipose organ is characterized by its function to balance energy homeostasis in response to various environmental stimuli. Depending on the situation, an excess of nutrients leads to energy storage in adipocytes in the form of triglycerides, while food deprivation leads to a release of fatty acids from the adipose triglyceride pool. The adipose organ comprises different adipose depots and fat cell types. The subcutaneous and visceral adipose tissue predominantly consists of energy storing white adipocytes, while energy-dissipating, thermogenic brown adipocytes are localized in the interscapular region in mice, while mainly located in the supraclavicular region in adult humans [95,96,97,98,99,100]. More recently, further studies have illustrated that inducible brown-like adipocytes also appear in white adipose depots upon cold exposure or beta-adrenergic stimulation, so-called brown-in-white (brite) or beige adipocytes. A temperature decline triggers norepinephrine release from the sympathetic nervous system (SNS), which leads, in a short-term fashion, to the activation of the already existing brown/brite adipocytes and, in a long-term fashion, to the recruitment of brite/brown adipocytes [101]. Activated brown adipocytes have been characterized as able to take up glucose to a very large degree, as demonstrated by PET/CT imaging. For their high metabolic capacity, thermogenic adipocytes are rich in mitochondria, which are able to uncouple the respiratory chain from ATP synthesis via the uncoupling protein 1 (UCP1), which allows to dissipate the proton gradient across the mitochondrial membrane for heat production instead of ATP synthesis, also called non-shivering thermogenesis [102]. As brite and brown adipocytes are able to massively combust energy and diminish blood glucose levels, they currently attract great attention as promising targets to ameliorate lipid and glucose metabolism in diabetes and obesity. 

lncRNAs have in general been identified to be important regulators in adipogenesis and adipocyte metabolism [49]. In the past four years, the role of lncRNAs in adipogenesis has been extensively studied. Historically, the first lncRNA, which was associated with adipogenesis is steroid receptor RNA activator (SRA), which was correlated to adipogenesis in murine 3T3-L1 adipocytes, partially as co-activator of peroxisome proliferator-activated receptors γ 2 (PPARγ2) [103]. lncRNA PU.1 AS has been shown to promote adipogenesis by regulating the adjacent mRNA Pu.1 and preventing its transcription [104,105]. The lncRNA NEAT1 has been described to be associated with the splicing regulating protein SRp40 to regulate PPARγ2 splicing during adipogenesis [106]. Interestingly, miR-140 has been shown to physically interact in the nucleus with NEAT1, leading to increased NEAT1 expression and promotion of adipogenesis [107]. The lncRNA ADINR, significantly overexpressed at the beginning of differentiation of human mesenchymal stem cells (hMSCs), is encoded about 450 basepairs upstream of CCAAT/enhancer-binding protein α (Cebpα), a transcription factor and key driver of adipocyte differentiation, and impacts H3K4me3 and H3K27me3 histone modifications at the Cebpα locus [108]. More recently, the lncRNAs ADNCR and H19 have also been implicated in adipogenesis but via different modes of action. lncRNA ADNCR decoys miRNA-204, an inhibitor of SIRT1 that is known to inhibit adipocyte differentiation via repressing PPARγ activity [109]. H19 reduces expression of class II Hdacs 4, 5, and 6, which are important factors in adipogenesis. Following a feedback regulatory loop, Hdac inhibition also reduced H19 expression [110]. Moreover, the adipose tissue enriched lncRNA U90926 has been shown to promote 3T3-L1 adipocyte differentiation via promoter transactivation of Pparγ2, but not of Cebpα. Nevertheless, Cebpα mRNA expression, as well as Fabp4 and AdipoQ, was affected by overexpression or inhibition of the lncRNA. In addition, lncRNA U90926 has been found to be elevated in expression in adipose tissues of obese mice [111]. The lncRNA MIR31HG has also been associated to adipocyte differentiation, as its overexpression promoted adipocyte differentiation and increased mRNA and protein expression of adipogenic factors, such as Pparγ, Cebpα, and fatty acid binding protein 4 (Fabp4), but was not sufficient to induce intracellular lipid accumulation without adipogenic supplements. In vivo, MIR31HG promoted de novo formation of adipose tissue. The authors found a mechanism by which MIR31HG enriched AcH3 and H3K4me3 in the promoter area of Fabp4 [112]. Moreover, the expression of lncRNA MEG3 was shown to be upregulated by pioglitazone to protect endothelial progenitor cells via decreasing miR-140-5p levels and increasing Hdac7 expression in subjects with metabolic syndrome [113]. MEG3 has also been shown to govern the balance between adipogenic and osteoblastogenic differentiation in human adipose-derived stem cells (hASCs) [114].

From a metabolic point of view, it is of great interest to pay attention to lncRNAs’ regulatory functions in thermogenic adipocytes. Only four lncRNAs have been described as regulators of thermogenic adipocyte differentiation. The first described lncRNA candidate was BLNC1, which forms a ribonucleoprotein complex with the transcription factor Ebf2, thus promoting brown and brite differentiation and function [115]. In addition, another lncRNA, hnRNPU, acts as an RNA-binding protein, which stabilizes the ribonucleoprotein complex BLNC1/Ebf2 assembly, thus increases its transcriptional function [116]. Here, the mode of action to the lncRNA regulation has also been elucidated in which the zinc finger and BTB domain-containing 7b (Zbtb7b) protein recruits the lncRNA BLNC1/hnRNPU complex to activate thermogenic gene expression [117]. In another study, a comparative transcriptional analysis of the three different murine adipose tissues revealed 127 lncRNAs with an expression pattern restricted to BAT that are targeted in their promoter region by Cebpα, Cebpβ, and Pparγ. Among those, the lncRNA BATE1 has been directly connected to brown adipocyte differentiation. BATE1 is enriched 10–20-fold during brown adipogenesis and is essential for establishing the gene program necessary for both brown adipocyte development and later functional maintenance. lncRNA BATE1 acts in *trans* and binds hnRNPU and is an inducer of several brown markers, such as Dio2, Elovl3, Pparα, and Ucp1, as well as for the common adipogenic factors Cebpα and Pparγ [118]. Very recently, the same team also found a link between BATE1 and BATE10, another brown adipose tissue-enriched lncRNA [119]. Upon browning via cold exposure, CL316243 treatment, or intensive swimming training of mice, BATE1 appeared to be upregulated during brown adipocyte differentiation and induced 7-fold upon BAT activation. lncRNA BATE10 was regulated by the cAMP-Creb signaling pathway and interacted with CUG-binding Protein (Celf1) to finally compete with Pgc1α for Celf1 binding. Celf1 is known to bind the 3′-UTR of its target mRNAs to promote RNA degradation and to repress translation. By competing with Celf1, lncRNA BATE10 impaired its inhibitor effect on Pgc1α and promoted brown adipocytes differentiation [119].

Brown adipocytes are important regulatory determinants in energy homeostasis, and also here lncRNAs have an important regulatory impact. In addition to the regulation by transcription factors, epigenetic modifications operated by lncRNAs might be a missing keystone to the tremendous plasticity of the adipose organ.

## 3. Conclusions

lncRNAs constitute the most abundant part of the mammalian transcriptome. They have been shown to have an impact on all essential processes in the living cells including replication, chromatin shaping, transcription, splicing, translation, and posttranslational modification of proteins. As all nascent molecules of interest, lncRNA research is facing a great number of challenges. One challenge is that—in contrast to protein-coding genes—lncRNAs are poorly conserved between species. Another one is that lncRNAs exert their functions via multiple mechanisms, in different cellular compartments, with various binding partners such as proteins, mRNAs, microRNAs, and lncRNAs, thus adding another regulatory layer and complexity to the regulatory network that we know so far. In addition, a unique classification system and nomenclature for lncRNAs is still missing. A sound insight into the mode of action of each individual lncRNA would allow us to assign them to regulatory pathways. To further facilitate the knowledge discovery and retrieval, a systematic classification and nomenclature as well as one comprehensive database for lncRNAs—such as miRBase for microRNAs—would be a great contribution and support for the scientific community. 

In addition to the physiologic impact of lncRNAs in metabolic organs, their dysregulation is also implicated in metabolic diseases. Thus, modulation of lncRNA expression holds a promising potential for therapeutic applications [120], and due to the fact that lncRNAs can also circulate via exosomes—maybe as part of the inter-organ crosstalk—they also provide the opportunity to be used as biomarkers for diagnostic applications [121]. 

**Figure 1:**
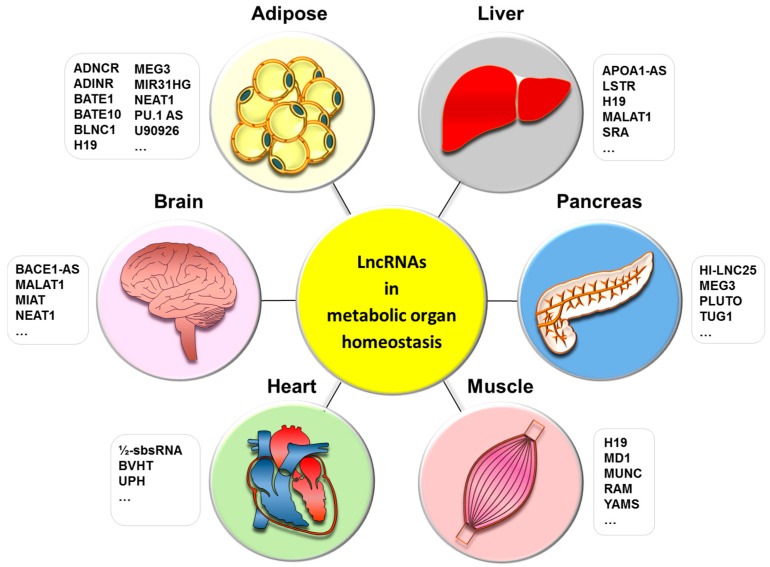
LncRNAs in metabolic organ homeostasis. This figure represents the metabolic organs implicated in energy homeostasis and their associated lncRNAs; in liver: lncRNA APOA1-AS, lncRNA H19, lncRNA LSTR, lncRNA MALAT1, lncRNA SRA; in pancreas: lncRNA HI-LNC25, lncRNA MEG3, lncRNA PLUTO, lncRNA TUG1; in skeletal muscle: lncRNA H19, lncRNA MD1, lncRNA RAM, lncRNA YAM-1; in heart: lncRNA 1/2-sbsRNA B2, lncRNA BVHRT, lncRNA UPH; in brain: lncRNA BACE1-AS, lncRNA MALAT1, lncRNA MIAT, lncRNA NEAT1; in adipose tissue: lncRNA ADINR, lncRNA ADNCR, lncRNA BATE1, lncRNA BATE10, lncRNA BLNC1, lncRNA H19, lncRNA MEG3, lncRNA MIR31HG, lncRNA NEAT1, lncRNA PU.1 AS, lncRNA U90926.

In the context of metabolic control, we summarized in this review a broad range of lncRNAs in metabolic tissues that may affect energy homeostasis, and described their direct targets if available (Table 1, Figure 1). 

It is interesting to see that most of the lncRNAs related to metabolic organs are described as tissue-specific. However, lncRNAs such as H19, MIAT, and NEAT1 seem to be shared by different organs. That might be explained by the fact that these lncRNAs have been studied more extensively and in different models or that they play more essential and general roles in all cells. Moreover, there are numerous lncRNAs that have been described as regulated between a lean and obese phenotype, in mice and humans, without further functional characterization. Moreover, circulating lncRNAs, lncRNA-p5549, lncRNA-p21015, and lncRNA-p19461, have been studied in lean and obese human subjects, as well as in obese patients submitted to diet for 12 weeks. It appeared that those three lncRNAs are inversely correlated with body mass index (BMI), waist circumference, waist-to-hip ratio, and fasting insulin levels [122]. This observation provides evidence that lncRNA synthesis and secretion can be also responsive to energetic states of the body. In mice, distinct lncRNA signatures have been described in three adipose tissues upon cold exposure, among them BATE-1, BATE-10, and BLNC1 were directly related to browning [115,116,117,118,119,122]. In this review, we have summarized lncRNAs and their functions in metabolic organs and energy homeostasis responsive to endogenous and exogenous stimuli, including candidate lncRNAs with an impact on disease states. Nevertheless, we are still a long way from establishing lncRNAs as a therapeutic target or agent for the amelioration of metabolic disorders. This is due to the lack of functional and mechanistic characterization of many lncRNA candidates, including their side effects. In comparison to the substantial number of lncRNA loci in the mammalian genome, our knowledge about lncRNAs in metabolic organs and energy homeostasis is still in its infancy. However, on the other side of the coin, the rest of the iceberg is a treasure chest yet to be discovered. 

## Figures and Tables

**Table 1:** Long non-coding RNAs (lncRNAs) in metabolic organs.

Organ	LncRNA	Biological Context	Physiological Context	Direct Target	Species	Ref.
**Liver**	lncRNA APOA1-AS	Reverse cholesterol transport	Hepatic physiology	H3K4-met3, H3K27-met3	human/monkey	[52]
	lncRNA LGR	GCK expression, glycogen storage	Lipid metabolism dysregulation	hnRNPL	mice	[55]
	lncRNA LSTR	Triglyceride levels	Lipid homeostasis in liver	Tdp-43	mice	[51]
	lncRNA MALAT1	Hepatic steatosis, insulin resistance	Diabetes	Srebp-1c	mice	[54]
	lncRNA SRA	Hepatic steatosis	Hepatic steatosis	FoxO1	mice	[53]
	lncRNA H19	Gluconeogenesis	Hepatic physiology	FoxO1	mice	[56]
**Pancreas**	lncRNA HI-LNC25	Endocrine cells: specification and function	Diabetes	Glis3	mice	[61]
	lncRNA MEG3	Insulin synthesis and secretion	Diabetes	Pdx-1, MafA	mice	[64]
	lncRNA PLUTO	Glucose tolerance	Diabetes	Pdx1	human	[63]
	lncRNA TUG1	Insulin synthesis and secretion	Diabetes	-	mice	[62]
**Skeletal muscle**	lncRNA DBE-T	Polycomb/trithorax epigenetic switch	FSDH muscular dystrophy	Ash1L	human	[80]
	lncRNA H19	Differentiation	Myogenesis	miR-675-3p, miR-675-5p	mice	[77]
	lncRNA MD1	Differentiation	Myogenesis	HuR	mice	[79]
	lncRNA MD1	Differentiation	Myogenesis	miR-133, miR134	mice	[78]
	lncRNA MUNC	Differentiation	Myogenesis	MyoD	mice	[75]
	lncRNA RAM	Differentiation of satellite cells	Myogenesis	MyoD	mice	[81]
	lncRNA YAM-1	Differentiation	Myogenesis	miR-715, Wnt7b	mice	[76]
**Cardiac muscle**	lncRNA 1/2-sbsRNA B2	Differentiation	Differentiation	Traf6	mice	[87]
	lncRNA APF	Autophagy, myocardial cell death	Cardiovascular diseases	miR-188-3p	mice	[47]
	lncRNA BVHRT	Development	Cardio vascular diseases	Suz12	mice	[85]
	lncRNA CARL	Apoptosis	Cardiovascular diseases	miR-539	mice	[47]
	lncRNA CHAER	Hypertrophy	Cardiovascular diseases	Prc2	human/mice	[47]
	lncRNA CHRF	Hypertrophy	Cardiovascular diseases	miR-489	human/mice	[47]
	lncRNA H19	Hypertrophy	Cardiovascular diseases	miR-675	mice	[47]
	lncRNA H19	Necrosis	Cardiovascular diseases	miR-103/107	mice	[47]
	lncRNA MHRT	Hypertrophy	Cardiovascular diseases	Brg1	human/mice	[47]
	lncRNA MIAT	Hypertrophy	Cardiovascular diseases	miR-150	rat	[47]
	lncRNA NRF	Necrosis	Cardiovascular diseases	miR-873	mice	[47]
	lncRNA p21	Proliferation and apoptosis	Cardiovascular diseases	Mdm2	human/mice	[47]
	lncRNA ROR	Hypertrophy	Cardiovascular diseases	miR-133	mice	[47]
	lncRNA UPH	Development	Cardio vascular diseases	Gata4, histone acetylation	mice	[86]
**Brain**	lncRNA BACE1-AS	αβ peptide production	Alzheimer’s disease	BACE1-AS	human/mice	[93]
	lncRNA MIAT	Brain development	Brain development	Wnt7b	mice	[90]
	lncRNA NEAT1	Synapse formation	Huntington disease	FUS/TD-43	human/mice	[91]
**Adipose organ**	lncRNA U90926	White adipogenic differentiation	Obesity	Pparγ2 and Pparγ	mice	[111]
	lncRNA ADINR	White adipogenic differentiation	Obesity	Pa1	human	[108]
	lncRNA ADNCR	White adipogenic differentiation	Obesity	miR-204	bovine	[109]
	lncRNA BATE1	Brown adipogenic differentiation	Energy homeostasis	hnRNP U	mice	[118]
	lncRNA BATE10	Brown adipogenic differentiation	Energy homeostasis	Celf1	mice	[119]
	lncRNA BLNC1	Brown adipogenic differentiation	Energy homeostasis	Zbtb7b	mice	[117]
	lncRNA BLNC1	Brown adipogenic differentiation	Energy homeostasis	hnRNPU	human/mice	[116]
	lncRNA BLNC1	Brown adipogenic differentiation	Energy homeostasis	Ebf2	mice	[115]
	lncRNA H19	White adipogenic differentiation	Obesity	class II Hdacs 4,5,6	human	[110]
	lncRNA MEG3	Adipogenic/osteogenic differentiation	Obesity	miR-140-5p	human	[114]
	lncRNA MEG3	Endothelial progenitor cells differentiation	Metabolic syndrome	Hdac7/miR-140-5p	human	[113]
	lncRNA MIR31HG	White adipogenic differentiation	Adipogenesis	Fabp4	human	[112]
	lncRNA NEAT1	White adipogenic differentiation	Obesity	miR-140	mice	[107]
	lncRNA NEAT1	White adipogenic differentiation	Obesity	SRp40	mice	[106]
	lncRNA PU.1 AS	White adipogenic differentiation	Obesity	Pu.1	mice/pig	[104,105]
